# Semi-Supervised Deep Learning Semantic Segmentation for 3D Volumetric Computed Tomographic Scoring of Chronic Rhinosinusitis: Clinical Correlations and Comparison with Lund-Mackay Scoring

**DOI:** 10.3390/tomography8020059

**Published:** 2022-03-07

**Authors:** Chung-Feng Jeffrey Kuo, Yu-Shu Liao, Jagadish Barman, Shao-Cheng Liu

**Affiliations:** 1 Department of Materials Science & Engineering, National Taiwan University of Science and Technology, Taipei 114, Taiwan, jeffreykuo@mail.ntust.edu.tw (C.-F.J.K.); jeffrey6349754@gmail.com (Y.-S.L.); D10904820@mail.ntust.edu.tw (J.B.); 2 Department of Otolaryngology-Head and Neck Surgery Tri-Service General Hospital, National Defense Medical Center No. 325, Sec. 2, Cheng-Gong Road, Neihu District, Taipei 114, Taiwan

**Keywords:** three-dimensional CT, Lund-Mackay score, artificial intelligence, semi-supervised deep learning, MobileNet, SENet, ResNet

## Abstract

Background: The traditional Lund-Mackay score (TLMs) is unable to subgrade the volume of inflammatory disease. We aimed to propose an effective modification and calculated the volume-based modified LM score (VMLMs), which should correlate more strongly with clinical symptoms than the TLMs. Methods: Semi-supervised learning with pseudo-labels used for self-training was adopted to train our convolutional neural networks, with the algorithm including a combination of MobileNet, SENet, and ResNet. A total of 175 CT sets, with 50 participants that would undergo sinus surgery, were recruited. The Sinonasal Outcomes Test-22 (SNOT-22) was used to assess disease-specific symptoms before and after surgery. A 3D-projected view was created and VMLMs were calculated for further comparison. Results: Our methods showed a significant improvement both in sinus classification and segmentation as compared to state-of-the-art networks, with an average Dice coefficient of 91.57%, an MioU of 89.43%, and a pixel accuracy of 99.75%. The sinus volume exhibited sex dimorphism. There was a significant positive correlation between volume and height, but a trend toward a negative correlation between maxillary sinus and age. Subjects who underwent surgery had significantly greater TLMs (14.9 vs. 7.38) and VMLMs (11.65 vs. 4.34) than those who did not. ROC-AUC analyses showed that the VMLMs had excellent discrimination at classifying a high probability of postoperative improvement with SNOT-22 reduction. Conclusions: Our method is suitable for obtaining detailed information, excellent sinus boundary prediction, and differentiating the target from its surrounding structure. These findings demonstrate the promise of CT-based volumetric analysis of sinus mucosal inflammation.

## 1. Introduction

One of the most widely used computed tomography (CT)-based scoring systems for chronic rhinosinusitis (CRS) is the Lund-Mackay system (LMs) [1]. With scores ranging from 0–24, it provides a simple technique with semi-quantitative analysis. This system has been lauded for its low inter-observer variability that makes for quick, competent use by those without formal radiology training [2]. Despite its popularity and ease of use, the LMs lags behind in sensitivity to change and does not correlate strongly with patient symptoms nor with quality of life [3,4], likely due to its inability to distinguish varying degrees of partial opacification. Various modifications have been made to further stratify the grade levels [5], so as to achieve finer resolutions, but this has led to a lower inter-observer agreement and decreased its facility for applicability.

Since the main drawback of the traditional LMs (TLMs) is its inability to “subgrade” the volume of inflammatory disease, recent studies have focused on creating an objective scoring system by utilizing software-based tools and three-dimensional (3D) measurement of sinus inflammation using volumetric approaches [6]. Convolutional neural networks (CNN)—one of the primary data processing models used in deep learning, a subfield of artificial intelligence—have emerged as powerful tools for automatic medical image analysis. Even though these modern scoring methods show promising results, all of them are tailored to classic CNN architectures and are often only examined on small-scale computer vision datasets. Meanwhile, existing segmentation methods rely on manual or semiautomatic segmentation of the sinus cavities [7]. To train deep neural networks, large amounts of labeled data are usually necessary. In the medical field, however, labeled data is scarce, as manual annotation is time consuming and tedious. At the same time, when training models use a limited amount of labeled data, there is no guarantee that these models will generalize well on unseen data that is distributed slightly differently.

Semi-supervised learning may provide a means to leverage both a limited amount of labeled data and arbitrary amounts of unlabeled data to train deep networks [8]. At present, the networks commonly used in medical image segmentation include full CNN (FCN), PSPNet, and DeepLab-V3+. However, research on the use of semantic segmentation networks to segment the paranasal sinuses is still rare [9]. This paper proposes a semi-supervised and automatic segmentation algorithm by combining MobileNet, the squeeze-and-excitation networks (SENet), and ResNet. The first goal of this study was to validate the result by comparing our processing with state-of-the-art approaches. The secondary objective was to apply our algorithm to assess sinus inflammation by calculating the mucosa-to-sinus volume ratio and the modified LM score through 3D CT analysis. We hypothesize that the computerized, volume-based, modified LM score (VMLMs) would correlate more strongly with clinical symptoms than the visual, subjective TLMs, which could affect clinical decision making and guide medical or surgical treatment.

## 2. Materials and Methods

### 2.1. Clinical Metrics and CT Annotation

Just prior to CT imaging with 1 mm contiguous sections, patients were asked to complete a validated survey, namely the Sinonasal Outcomes Test-22 (SNOT-22) [10], which measures disease-specific quality of life; this survey was repeated 3 months later for patients that underwent surgery for CRS. The present patient cohort included those that received a sinus CT scan because of a suspected diagnosis of CRS. Some of the CT images (around 28%) were annotated for CNN training, and each sinus was manually outlined and labeled by the same board-certified rhinologist. All outlines were independently reviewed for accuracy by three trained observers (two otolaryngologists and one radiologist). The other CT images were reserved for validation and testing.

### 2.2. Semi-Supervised Learning

Pseudo labels are artificial labels generated by semi-supervised learning that use the labeled data to train the CNN first. By minimizing the entropy for the unlabeled data, the overlap of the class probability distribution was reduced, and we chose the class with the maximum predicted probability every weight updated as the pseudo labels. Pseudo labels were used as if they were true labels and to further train the model with a larger dataset. However, predictions on the unlabeled data were not always correct, and we had to filter them based on a confidence threshold. Meanwhile, by exploiting the unlabeled data, we also modeled the per-sample loss distribution with a mixture model to dynamically divide the training data into a labeled set with clean samples and an unlabeled set with noisy samples and trained the model on both the labeled and unlabeled data in a semi-supervised manner. After iterating this process, this training method demonstrated substantial improvements over state-of-the-art methods.

### 2.3. Improved Semantic Segmentation Model

The job of the convolution layer was split into two subtasks: first, there was a depthwise convolution layer that filtered the input, followed by a 1 × 1 (or pointwise) convolution layer that combined these filtered values to create new features. Together, the depthwise and pointwise convolutions formed a “depthwise separable” convolution block, to replace the traditional convolutional layers, and that is the main idea behind MobileNet.

To strengthen the representational power of the CNN by enhancing the quality of spatial encodings throughout its feature hierarchy, we used SENet to adaptively recalibrate channel-wise feature responses by explicitly modeling interdependencies between channels. SENet can learn to use global information to selectively emphasize informative features and suppress less useful ones. The activation function used in SENet was changed from ReLU to Mish, which can prevent activations from becoming too big [11]. The final output of the block was obtained by rescaling the original output with the activation function, and the excitation operator referred to channel-wise multiplication between them.

Finally, we used ResNet (skip connection via addition) to backpropagate through the identity function, just by vector addition [12]. The gradient was simply multiplied by one and its value was maintained in the earlier layers. ResNet stacked the skip residual blocks together to solve the problem of the gradient vanishing during training in very deep neural networks.

### 2.4. D Volumetric Image Analysis

After each slice of the sinus was segmented, the total volume, volume of air, and volume of disease were calculated. The segmented slices were reconstructed into three-dimensional (3D) solids. The VMLMs were calculated by multiplying the mucosa-to-sinus volume ratio by 2 to match the range of values of the TLMs, and the results were summed to obtain the total VMLMs for each sinus. To obtain the TLMs, the coronal planes of the 2D CT slices were also segmented by our AI-automated method.

## 3. Statistical Analysis

Data were presented as the mean ± standard deviation. Student’s *t*-test, one-way ANOVA, and linear regression were used for statistical analysis. Differences were assumed significant at *p* < 0.05.

## 4. Ethical Considerations

The research protocol (NO: C202105070) was reviewed and approved by the Institutional Review Board.

## 5. Results

**Figure 1:**
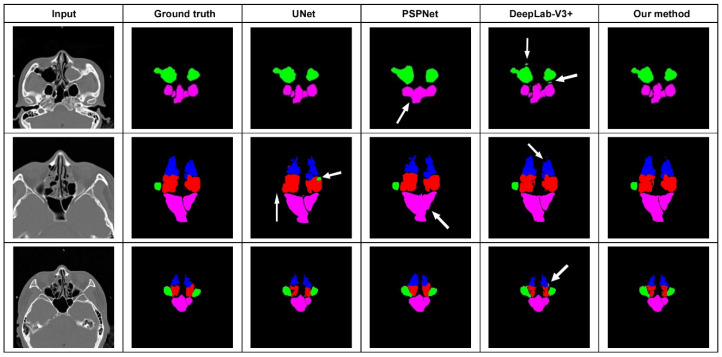
Segmentation results from comparison between our proposed method and other state-of-the-art networks. U-net misjudged part of the ethmoid sinus as the maxillary sinus. PSPnet had the least fine detailed information. Deeplab-V3+ showed inadequate accuracy in sinus boundary interpretation.

This study included 175 CT datasets, obtained from 111 men and 64 women, with a mean age of 49 (between 21 and 80) years. Fifty labeled sets were randomly split into training and validation sets according to a ratio of 8:2. Twenty percent (*n* = 25) of the 125 unlabeled sets were used for self-training, and actual testing was performed on the other 100 sets (100/125, 80%). Automatic sinus segmentation in the test cohort required about 0.082 s of computation time per scan and 9.73 s per set. The accuracy of sinus classification, as judged by 3 specialists, was 94.55 ± 4.15% (Table 1). Slightly inferior accuracies were noted for the anterior and posterior ethmoid sinuses (88.5~92.5%), while a high discrimination ability was proved for the other sinuses (>95%). Our method achieved better segmentation results, whose average Dice coefficient was 91.57 ± 2.17%, MIoU was 89.43 ± 3.56%, and pixel accuracy was 99.75 ± 2.84% (Table 2). As for the comparison with state-of-the-art methods, PSPnet had the least fine detailed information and worst sinus boundary prediction. U-net was poor in distinguishing the target from its surrounding structure, misjudging part of the ethmoid sinus as the maxillary sinus. Compared with the U-Net, our architecture increased the Dice efficiency and MIoU by 1.89% and 1.60%, respectively. Finally, Deeplab-V3+ showed inadequate accuracy in sinus boundary interpretation, and the output was blurry, especially for the maxillary and sphenoid sinuses. Our algorithm effectively reduced region misjudgment and improved the segmentation accuracy compared with U-Net, PSPnet, and Deeplab-V3+ (Figure 1).

**Figure 2:**
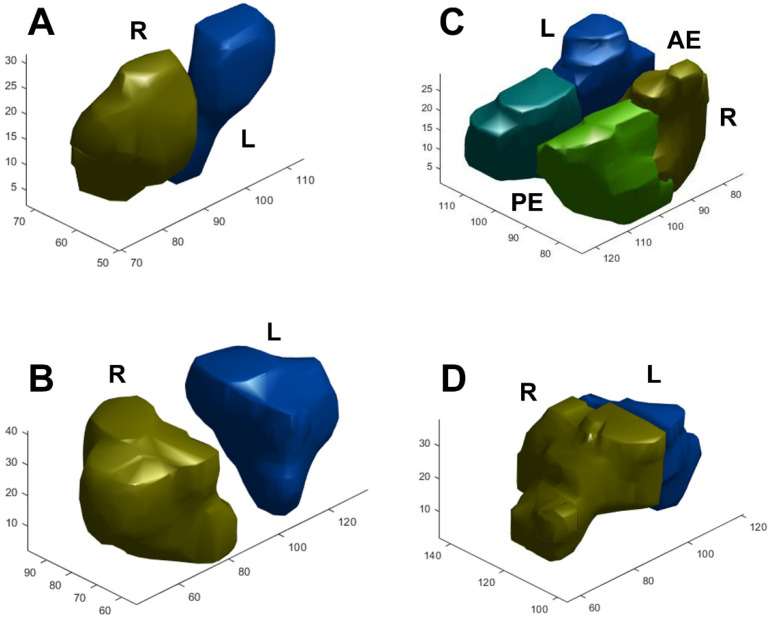
Examples of segmentation and 3D reconstruction of the (**A**) frontal, (**B**) maxillary, (**C**) anterior and posterior ethmoid, and (**D**) sphenoid sinus. R/L: right/left side, AE/PE: anterior/posterior ethmoid sinus.

**Figure 3:**
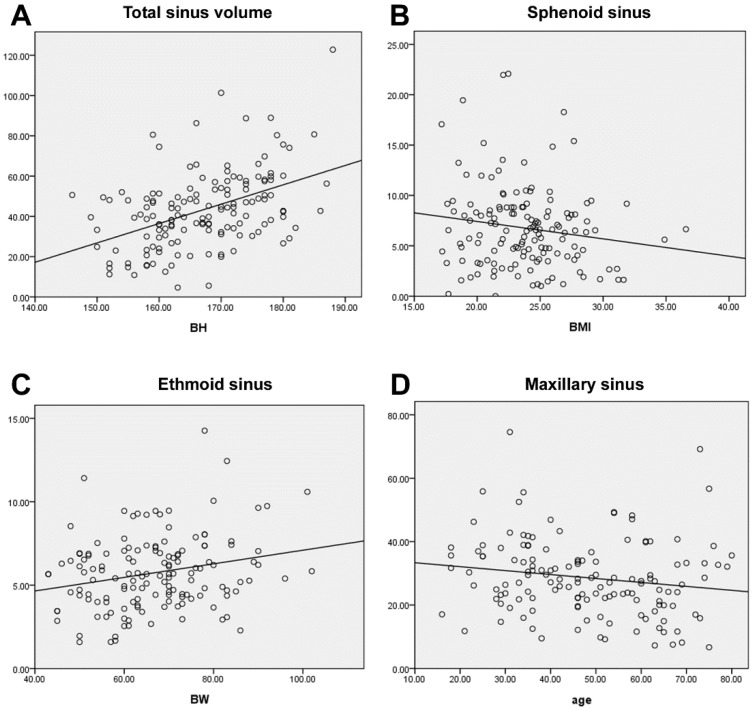
(**A**). A significant positive correlation was found between sinus volume and body height (*p* < 0.001). The volume had no significant correlations with BMI (**B**) (*p* = 0.067) and weight, except for the ethmoid sinus (**C**) (*p* = 0.005). (**D**) A trend toward negative relation between maxillary sinus volume and age (*p* = 0.053).

The average volume of the sinus cavities based on automatic segmentation (Figure 2) and the difference in volume according to sex are summarized in Table 3. Total sinus volumes ranged between 15.47 and 122.76 mL (mean = 44.9 mL). Correlations between single sinus volumes and age, sex, height, and weight differed. Each sinus volume in men was larger than the corresponding sinus volume in women, and men are typically taller and heavier than women. A significant positive correlation between each sinus volume and body height was found. This relationship remained true after multiple linear regression, controlling for age, sex, and weight (Figure 3A). As for sex dimorphism, the relationship could be proved only in the frontal sinus after multiple linear regression analysis, controlling for age and height. There were no correlations between sinus volume and body weight, except for the volumes of the anterior and posterior ethmoid sinuses, which showed a positive correlation with weight (Figure 3B). Significant correlations between sinus volumes and BMI were not found (Figure 3C). A trend toward a negative relationship between maxillary sinus volumes and age could be proved (*p* = 0.053) (Figure 3D). As for the other sinuses, no significant correlations with age were found. When comparing the left and right side, volumes of the maxillary and anterior and posterior ethmoid sinuses showed no significant differences, but an obvious asymmetry was found in the frontal and sphenoid sinuses (*p* < 0.05), as displayed in Table 4. Although the significance of this observation is not clear, the ability to calculate sinus volume automatically may be useful in future research.

**Figure 4:**
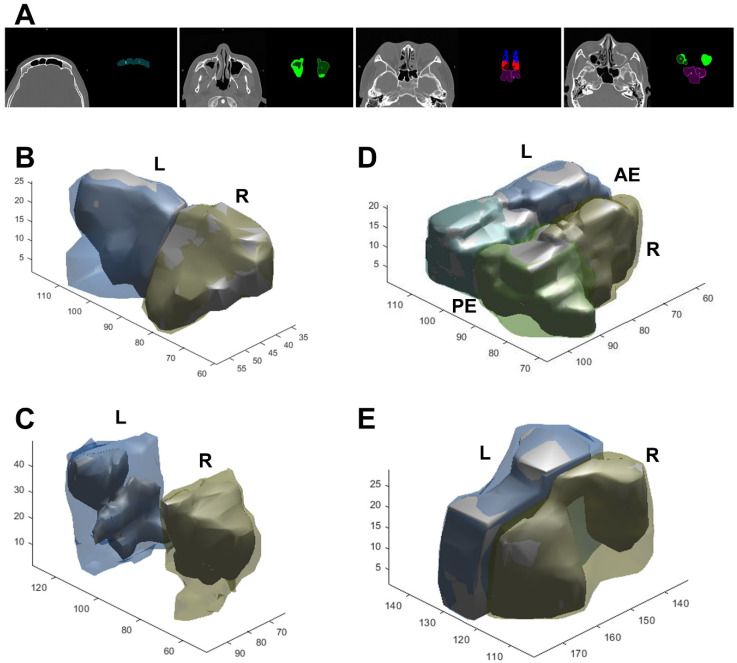
Examples of segmentation (**A**) and 3D reconstruction in CRS patients with air (inner solid part) and opacification (outer hallow part). (**B**) Frontal, (**C**) maxillary, (**D**) anterior and posterior ethmoid, and (**E**) sphenoid sinus. R/L: right/left side, AE/PE: anterior/posterior ethmoid sinus.

**Figure 5:**
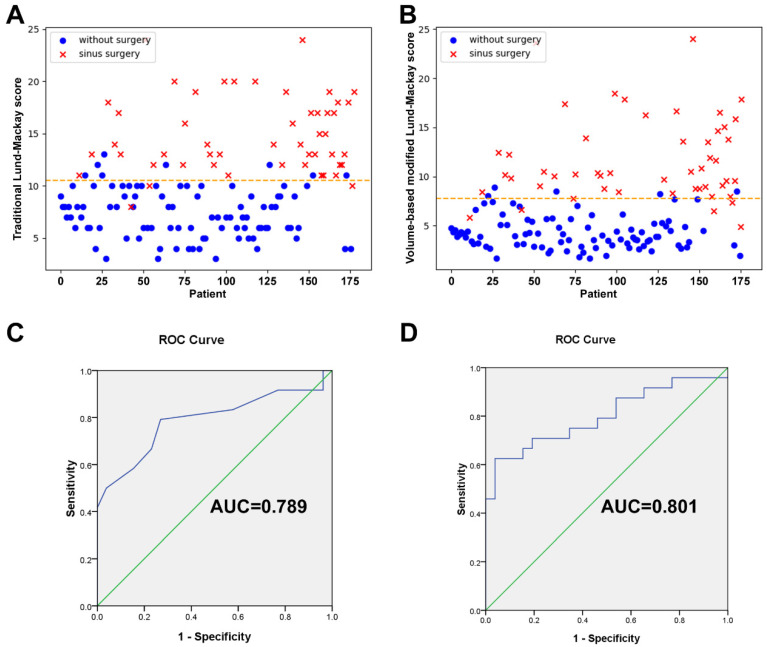
Scatter plots of (**A**) TLMs and (**B**) VMLMs values, grouped by whether. surgery was performed or not. ROC with AUC analysis for assessing the correlation between SNOT-22 improvement and the (**C**) TLMs and (**D**) VMLMs scoring system.

A 3D view projected directly from the volume data (volume rendering) was created and the opacification ratio was calculated for each sinus to obtain the VMLMs (Figure 4). The average VMLMs in the test cohort was 7.12, with an overall range of 1.98 to 24. Among 175 cases, 50 participants with established CRS underwent endoscopic sinus surgery. Subjects that underwent surgery had a significantly greater TLMs (14.9 vs. 7.38; *p* < 0.001) and VMLMs (11.65 vs. 4.34; *p* < 0.001) than those that did not (Table 5). Receiver operating characteristic (ROC) analysis showed that the cut-points for surgical intervention of TLMs and VMLMs were 10.5 and 7.75, respectively (Figure 5A,B). The median overall SNOT-22 scores before and 3 months after surgery were 42.38 (range 18–65) and 30.46 (range 15–45), respectively. A significant improvement in SNOT-22 was defined as a 25% reduction after surgery. ROC-AUC analyses were used to assess the discriminatory capability of the TLMs and VMLMs for SNOT-22 (Figure 5C,D). The AUC of the VMLMs was 0.801, which suggests that it had excellent discrimination in classifying a high probability for postoperative improvement, while the AUC of the TLM was 0.789, which shows acceptable discrimination. This indicates that our processing and the VMLMs were not only accurate but also had a higher correlation with symptom improvement.

## 6. Discussion

In recent years, deep learning has dominated medical image segmentation. The spatial pyramid pooling (SPP) module (e.g., PSPNet) [13] or the encoder–decoder structure (EDS) (e.g., U-Net) [14] are used in deep neural networks for semantic segmentation tasks. Various semantic segmentation models based on a pretrained CNN were proposed to extract the feature map and gather the contextual information of paranasal sinus CT scans. Humphries et al. [9] once used DenseNets to achieve automated calculation of sinus opacification. However, they only distinguished the entire sinuses from the nasal cavity and did not make a separate segmentation for each sinus. In this study, we compared our processing algorithm with other state-of-the-art approaches. We found that PSPNet has the worst semantic prediction of the sinus contour and yields uncertain predictions at the boundaries. To obtain multi-scale views of spatial contextual information, PSPNet performs SPP at several grid scales. This is an effective method, but PSPNet puts equal weights at every position and its final feature map size is 1/8 of the input image. The finely detailed information may be lost in the pooling operation, which can hamper the final performance of PSPNet. The compactness of the U-Net architecture has become the most time-consuming process in current research. U-Net includes a contraction path and an expanding path. It is basically an end-to-end FCN. U-Net puts emphasis on the calculation of context information in higher-resolution feature maps and combines it further with an up-sampled output. This computation results in a more precise output (as compared to FCN), but since many layers take a significant amount of time to train, a relatively high GPU memory footprint is needed. Meanwhile, U-Net has a small and fixed receptive field. Redundant features are extracted when the receptive field of the convolution kernel is too small. Smaller targets are ignored when the receptive field of the convolution kernel is too large. In our practice, we have observed that the edge detail of a smaller sinus is not fine when the receptor field is large and the structure of the sinus is not obvious when the receptor field is small. Therefore, it is very important to use a convolution kernel with different receptive fields to process the image. DeepLab-v3+ combines the advantages of the SPP module and the EDS. By introducing depthwise separable convolutions to both atrous SPP and decoder modules, DeepLab-v3+ can reduce the degree of signal down-sampling. However, DeepLab-v3+ begins with dimensionality reduction during down-sampling and discards the feature concatenation during up-sampling. Instead of using the skip connections that may help to retrieve detailed spatial information lost by pooling operations, as U-Net does, DeepLab-v3+ employs bilinear interpolation to perform up-sampling from small feature maps (1/4~1/8 of the input image). For biomedical image segmentation that focuses on the contour of the lesion, doing that will result in loss of the paranasal sinus shape details. The accuracy of the interpretation will be poor and the sinus boundaries will be blurred (Figure 1).

Compared to the above approaches, our architecture was augmented to expand the effective receptive field and calculate context information both in high-and low-resolution feature maps. Similar to DeepLab-V3+, MobileNet, which was used in this study, employs depthwise separable and pointwise convolutions for concatenation in up-sampled operators, which results in a faster and stronger network [15]. The dilated convolutions and atrous SPP can expand the receptive field that helps to further integrate information around the sinuses. To solve the problem of placing equal weights at every position by PSPNet, SENet was introduced with global average pooling to learn the influence of weights on each feature map, so as to further highlight the important information on it [16]. For up-sampling, we performed bilinear interpolation from a small (low-resolution) feature map and skipped connection by using ResNet, in order to propagate local information from an encoder path to a decoder path and retrieve detailed spatial information lost by pooling operations. The ResNet can avoid gradient degradation in the process of network deepening [17]. The contours of the sinuses became clearer and more precise after we fused different detailed features at different scales. It took approximately 0.082 s to segment a CT slice, and the average time consumption for the entire set of CT scan segmentation was 9.73 s, demonstrating that our algorithm was much more efficient than those used in previous studies [9,18]. In addition, our architecture increased the Dice coefficient to 91.57% and MIoU to 89.43%. Compared with U-Net, these parameters increased by 1.89% and 1.60%, respectively. When using our architecture, the doctor is not required to perform any manual operation in the whole segmentation process, which implies that segmentation is automatic and more efficient; this is very useful for finding the VMLMs, which gives the 3D volumetric blockage ratio.

The 3D morphological knowledge of paranasal sinuses has a primordial clinical value. It provides important information for the analysis of pathologies, planning of treatment strategies [19], monitoring of disease progression, and even for individual identification purposes [20]. For example, Wanzeler et al. determined the sex of subjects by analyzing their paranasal sinus volumes, achieving a high accuracy of 100% for 200 CT scans [21]. Significant differences in the frontal and maxillary sinus volumes according to gender were also reported [22]. In our study, we found that every sinus volume exhibited sexual dimorphism and that men had significantly larger sinuses than women. Multiple linear regression control and analysis showed that the true determinant of sinus volume was body height, although both sex and body height showed significant influence on the frontal sinus volume. As for weight, Ariji et al. [23]. found, in 115 CT scans, that the maxillary sinus volume was correlated with body weight in adult males. However, in our study, body weight had a significant impact only on the ethmoid sinus. Currently, there is no established consensus on age-related changes in sinus volume in adults, and it is believed that sinuses are hardly modifiable during life. Emirzeoglu found a weak negative correlation of maxillary sinus volume with age after the fourth decade [24]. Our study also reported a trend toward a reduction in maxillary sinus volume with age, which might be related to skeletal size and physique. The number of teeth might affect maxillary sinus volume in the elderly. As for the sidal difference (left/right), we found that the sphenoid and frontal sinuses were significantly more asymmetrical than the other sinuses. Indeed, recent clinical research studies have considered the existence of differences among individual paranasal sinuses and shown particularly that the frontal and sphenoid sinuses are anatomically extremely variable and unique to each individual [18,20]. Sizes, shapes, and pneumatization types vary from one person to another, even between twins, which could contribute to radiologic identification. This is extremely valuable for forensic identification purposes [21].

One major contribution of this study is the demonstration of the potential utility of volumetric assessment for the staging of sinus disease. Pallanch et al. [7] showed that volumetric quantification of sinus opacification on sequential CT outperformed TLMs when evaluating radiologic improvement after therapy. Likness et al. [25] compared multiple visual CT scoring systems with volumetric scoring based on manual CT segmentation and found that volumetric analysis was more sensitive to therapeutic effect. Although their results were promising, all of these efforts relied on manual or semiautomatic segmentation of the sinus cavities, which can take anywhere from 20 min to several hours to accomplish [26]. The current research, in which we identified the sinus boundary and calculated the opacification ratio automatically, was committed to solving this problem. VMLMs can be useful for providing a blueprint for treatment planning and for counseling of patients regarding the need for surgery. Through retrospective analysis of our surgical cases, we found that patients who had an average pre-operative TLMs of 14.9 or more and who failed maximal medical management were supposedly submitted to surgery, while those with TLMs less than 7.38 should undergo conservative treatment first. By using VMLMs, the average scores for surgery and conservative treatment were 11.65 and 4.34, respectively. ROC analysis showed that the cut-points for surgical intervention of TLMs and VMLMs were 10.5 and 7.75 respectively. This finding was similar to that of previous research, such as Singh et al. [27], who proposed that surgical intervention should only be considered in patients with a score of 6.55 or greater for good surgical outcomes. The interval scale of TLMs is coarse and two equally sized intervals on a TLMs scale are always interpreted as two equal disease severities. A complete 3D volumetric percentage of disease measurement of all sinuses could yield a numerical score of disease status on a continuous scale. Accordingly, VMLMs can interpret patients’ need for surgery more finely and accurately than TLMs. Compared to TLMs, efficient analysis of VMLMs revealed a better correlation between volumetric opacification scores and symptom improvement assessed using SNOT-22. Consequently, our finding is in agreement with those of a few studies in the medical literature [9,28] that suggest the inclusion of staging scores in routine sinus CT dictation. To our knowledge, the dataset of 175 patients used in this study is the largest cohort so far for a CRS study investigating volumetric image analysis. In the best interest of CRS patients, we propose the adoption of 3D volumetric computerized analysis of CT scans as the gold standard for measurement of disease extent.

Our study has some limitations. The dataset was obtained at a single institution, and just one measure of patients’ symptoms, SNOT-22, was used. The number of cases was not large and the improvement of patients’ symptoms was obtained by retrospective analysis and thus has some inherent limitations. In considering differences between CT equipment and imaging protocols, more studies should be carried out to test the reliability of our model in accommodating CT from other sites. Future work should include the development of customized software for user-specific applications, which will be incorporated into real-time evaluation.

## 7. Conclusions

The proposed approach achieves state-of-the-art performance on sinus segmentation. Fully automatic volumetric quantification of sinus opacification on CT provides an objective and reproducible method of measuring the extent of the disease in CRS and is very sensitive to change induced by treatment intervention. A better association with SNOT-22 would facilitate the selection of CRS patients who would benefit from surgery. Useful additional information can be provided for researchers and clinicians by incorporating this objective analysis into routine sinus CT evaluation.

## Figures and Tables

**Table 1:** Analysis agreement for our automatic segmentation result among three observers.

	Maxillary	AE	PE	Frontal	Sphenoid
Left	98%	88.5%	92.5%	95%	98.5%
Right	98.5%	88.5%	91%	96%	99.%

**Table 2:** Segmentation accuracy of sinus between three observers and automatic segmentation methods.

Methods	PA	Dice	MIoU
UNet	99.56%	89.68%	87.83%
PSPNet	99.31%	87.52%	85.78%
DeepLab-V3	99.55%	89.64%	87.69%
Ours	99.75%	91.57%	89.43%

**Table 3:** Mean values of the metrical characteristics and volume dispersion by sex, and results of the comparison between groups.

	Overall	Male	Female	*p*-Value	Male/Female
Age	47.88 ± 15.81	46.19 ± 16.35	51.16 ± 14.31	0.074	0.90
BH	167.07 ± 8.93	171.06 ± 7.64	159.33 ± 5.54	<0.01	1.07
BW	66.70 ± 12.58	71.16 ± 11.1	58.06 ± 10.71	<0.01	1.23
BMI	23.81 ± 3.63	24.30 ± 3.35	22.87 ± 3.98	0.024	1.06
Max-L	14.26 ± 6.12	15.39 ± 6.27	11.88 ± 5.06	<0.01	1.39
Max-R	14.39 ± 6.26	15.50 ± 6.45	12.04 ± 5.17	0.02	1.38
AE-L	1.49 ± 0.67	1.60 ± 0.72	1.28 ± 0.52	<0.01	1.25
AE-R	1.49 ± 0.56	1.57 ± 0.57	1.34 ± 0.50	<0.01	1.17
PE-L	1.35 ± 0.58	1.47 ± 0.57	1.13 ± 0.54	<0.01	1.30
PE-R	1.40 ± 0.61	1.50 ± 0.61	1.21 ± 0.57	<0.01	1.23
Fro-L	1.42 ± 1.24	1.68 ± 1.30	0.91 ± 0.92	<0.01	1.84
Fro-R	1.34 ± 1.23	1.64 ± 1.33	0.77 ± 0.69	<0.01	2.13
Sph-L	3.32 ± 2.00	3.64 ± 2.04	2.70 ± 1.77	<0.01	1.35
Sph-R	3.43 ± 2.35	3.81 ± 2.47	2.70 ± 1.90	<0.01	1.41

Test applied: Student’s *t*-test. R: right side, L: left side, Max: maxillary sinus, AE: anterior ethmoid sinus, PE: posterior ethmoid sinus, Fro: frontal sinus, Sph: sphenoid sinus.

**Table 4:** Sidal difference (left/right) subdivided between genders (one-way ANOVA). Max: maxillary sinus, AE/PE: anterior/posterior ethmoid sinus, Fro: frontal, Sph: sphenoid sinus.

Left/Right	Overall	Male	Female
Max	101.69 ± 19.76	102.24 ± 21.13	100.51 ± 16.95
AE	99.46 ± 18.83	101.48 ± 18.62	95.53 ± 18.81
PE	107.69 ± 21.76	107.01 ± 22.39	109.03 ± 20.31
Fro	188.68 ± 61.06	180.19 ± 59.92	206.01 ± 63.06
Sph	114.43 ± 69.47	113.70 ± 71.00	115.86 ± 67.11

**Table 5:** Opacification scores based on the TLMs and VMLMs stratified by surgical intervention. TLMs: traditional Lund-Mackay score; VMLMs: volume-based modified Lund-Mackay score. Max: maxillary sinus, AE/PE: anterior/posterior ethmoid sinus, Fro: frontal sinus, Sph: sphenoid sinus; OMC: ostiomeatal complex.

	Surgery (+)				Surgery (−)			
	TLMs		VMLMs		TLMs		VMLMs	
Total	14.9 ± 3.66		11.65 ± 4.23		7.38 ± 2.36		4.34 ± 1.73	
	Left	Right	Left	Right	Left	Right	Left	Right
Max	1.36 ± 0.72	1.56 ± 0.64	1.11 ± 0.72	1.32 ± 0.73	0.53 ± 0.67	0.56 ± 0.66	0.40 ± 0.50	0.37 ± 0.46
AE	1.7 ± 0.46	1.64 ± 0.48	1.32 ± 0.44	1.33 ± 0.45	1.04 ± 0.20	1.06 ± 0.25	0.68 ± 0.19	0.69 ± 0.23
PE	1.26 ± 0.49	1.22 ± 0.46	0.85 ± 0.50	0.77 ± 0.44	1.00 ± 0.29	1.02 ± 0.21	0.47 ± 0.25	0.47 ± 0.21
Fro	1.3 ± 0.54	1.34 ± 0.59	0.94 ± 0.69	1.02 ± 0.71	0.67 ± 0.47	0.70 ± 0.50	0.27 ± 0.14	0.31 ± 0.21
Sph	0.78 ± 0.65	0.66 ± 0.66	0.47 ± 0.52	0.44 ± 0.49	0.21 ± 0.41	0.23 ± 0.43	0.17 ± 0.10	0.17 ± 0.08
OMC	0.88 ± 1	1.2 ± 0.99	0.88 ± 2.01	1.2 ± 1.98	0.19 ± 0.59	0.15 ± 0.53	0.19 ± 1.18	0.15 ± 1.06

## Data Availability

The data presented in this study are available on request from the corresponding author.
